# Homogeneity and Thermal Stability of Sputtered Al_0.7_Sc_0.3_N Thin Films

**DOI:** 10.3390/ma16062169

**Published:** 2023-03-08

**Authors:** José Manuel Carmona-Cejas, Teona Mirea, Jesús Nieto, Jimena Olivares, Valery Felmetsger, Marta Clement

**Affiliations:** CEMDATIC-ETSI Telecomunicación, Universidad Politécnica de Madrid, Av. Complutense 30, 28040 Madrid, Spain

**Keywords:** AlScN films, 30% Sc-doped AlN, reactive magnetron sputtering, temperature stability, FBAR, BAW devices

## Abstract

This work presents a study on the homogeneity and thermal stability of Al_0.7_Sc_0.3_N films sputtered from Al-Sc segmented targets. The films are sputtered on Si substrates to assess their structural properties and on SiO_2_/Mo-based stacked acoustic mirrors to derive their piezoelectric activity from the frequency response of acoustic resonators. Post-deposition annealing at temperatures up to 700 °C in a vacuum are carried out to test the stability of the Al_0.7_Sc_0.3_N films and their suitability to operate at high temperatures. Despite the relatively constant radial composition of the films revealed from RBS measurements, a severe inhomogeneity in the piezoelectric activity is observed across the wafer, with significantly poorer activity in the central zone. RBS combined with NRA analysis shows that the zones of lower piezoelectric activity are likely to show higher surface oxygen adsorption, which is attributed to higher ion bombardment during the deposition process, leading to films with poorer crystalline structures. AFM analysis reveals that the worsening of the material properties in the central area is also accompanied by an increased roughness. XRD analysis also supports this hypothesis, even suggesting the possibility of a ScN non-piezoelectric phase coexisting with the AlScN piezoelectric phase. Thermal treatments do not result in great improvements in terms of piezoelectric activity and crystalline structure.

## 1. Introduction

Piezoelectric materials play an important role within critical industries today, such as telecommunications and sensors industries. These materials form the core of electroacoustic resonators, which are essential to the development of RF filter technology. High piezoelectric activity means good communication speed and larger bandwidths, a requirement needed to reach 5G standards and help establish this technology as the new telecommunications benchmark [1]. Therefore, studying magnitudes such as the electromechanical coupling coefficient (k^2^) becomes mandatory as higher k^2^ values tell us that the resonators fabricated with these materials could provide wider bandwidths working as RF filters [2]. In addition to this, when the appropriate trade-off between k^2^ and quality factor (Q-factor) values is reached, these acoustic resonators are suitable for other applications, already under study for several years, such as sensing applications. For instance, their surface can be functionalized to chemically detect several species and act as gravimetric sensors. This has been proven to work in both gaseous [3] and liquid environments [4]. These devices can also be suitable to operate at high temperatures, provided their properties remain stable under harsh environments [5].

One of the most studied and used piezoelectric material is AlN, which has plenty of advantages, such as a mature fabrication technology, either for solidly mounted resonators (SMRs) or free-standing film bulk acoustic resonators, and production steps well implemented in industry [6]. Other materials, such as ZnO [7], BTO [8,9] and PZT [10] have also been of use as piezoelectric materials in the RF filter industry for a long time. However, these materials might not have all the acoustic properties and piezoelectric qualities to reach 5G standards.

In recent years, new materials such as LiNbO_3_ [11] and AlScN [12] have come into play, offering good prospects for increasing piezoelectric activity to achieve broader bandwidths. Despite its very large electromechanical coupling factor, LiNbO_3_ poses serious manufacturing challenges when it comes to integrating it into acoustic resonators, mainly due the difficulty of growing LiNbO_3_ thin films with good crystalline properties at temperatures compatible with ongoing resonator technologies [13,14,15]. However, since Akiyama reported in 2009 that Sc-doped AlN films could provide an improved piezoelectric response [16], multiple researchers and companies have rushed to try to develop fabrication processes that can fit the manufacturing lines already used for AlN-based devices. Different thin film fabrication methods have been approached, such as molecular beam epitaxy [17] and co-sputtering with separate Al and Sc targets [18]. Although they have been proven to result in good film quality, these methods exhibit a few issues, as the former lacks high film growth rates and the latter presents difficulties in terms of production, as film homogeneity is hardly achievable on large substrates. Therefore, deposition methods such as the reactive magnetron sputtering of alloyed and/or engineered Al-Sc targets have become an interesting alternative to studying AlScN films when large production wafers are needed with enough piezoelectric quality and a uniform film composition. Post-deposition annealing of AlN films has been proven to be a helpful tool to obtain improved material quality and reduce film stress [19].

This work is an extension of our previous investigations on the growth of AlScN films sputtered from engineered Al targets containing embedded Sc pellets with concentrations up to 14% [20]. The goal of the present work is to help develop a sputtering process leading to uniform AlScN films with a 30% Sc content across 8″ substrates that could be, ultimately, transferred to industry. This requires a deep study of AlScN film properties across the wafer to try to link them to the different process parameters, which would allow feedback to be obtained for the deposition process. With this objective, we have assessed the piezoelectric, morphological and structural properties of Al_0.7_Sc_0.3_N films sputtered from an Al/Sc segmented target containing 30% Sc, paying a special attention to their uniformity across the wafer. The morphology, the surface topography and the crystalline structure of the films were assessed using scanning electron microscopy (SEM), atomic force microscopy (AFM) and X-Ray diffraction (XRD). Their composition was assessed using Rutherford backscattering spectrometry (RBS) and non-RBS (NRBS) measurements, at higher energies combined with nuclear reaction analysis (NRA). The piezoelectric activity of the films was derived from the frequency response of solidly mounted resonators (SMRs) containing the active layers. Post-deposition annealing of the samples at temperatures up to 700 °C were conducted to (1) investigate if the material properties and piezoelectric response could be improved, (2) obtain more insight about the quality of the films and thermal stability of the crystalline structure and (3) envisage possible high temperature applications.

## 2. Sample Preparation

**Figure 1:**
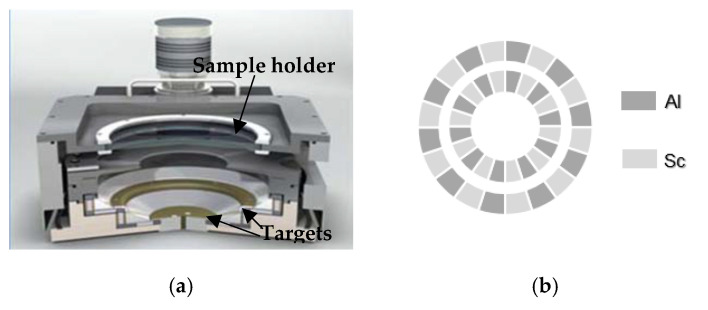
(**a**) Cross-sectional diagram of the sputtering chamber. (**b**) Coaxial Al-Sc segmented targets.

Al_0.7_Sc_0.3_N thin films were deposited in an Endeavor-MX PVD cluster tool from OEM Group LLC. The tool was equipped with an S-gun magnetron with a special target arrangement, consisting of two independently controlled ring-shaped coaxial sputtering targets of 7 and 11 inches in diameter, which were engineered to alternate Al and Sc segments (as displayed in Figure 1a,b). The size of the segments was set to achieve a 70/30 Al-to-Sc composition ratio. The target arrangement allowed the reactive sputtering of nitrides and oxides to be stable and arc-free, along with the ability to reach good compositional homogeneity in relatively large regions, as we will see in the next section. More insight on this topic can be found in [21]. Prior to film deposition, the ring-shaped targets were preconditioned, first in metallic mode under an Ar atmosphere, and then in poisoned mode with an Ar-N_2_ mixture. This last step prevented the films from being contaminated with Al_3_Sc precipitations. To ensure good film orientation and reduce roughness, a 50 nm AlN seed layer was first deposited on the substrates. Then, without breaking the vacuum, the AlScN films were deposited without intentional heating [22].

**Figure 2:**
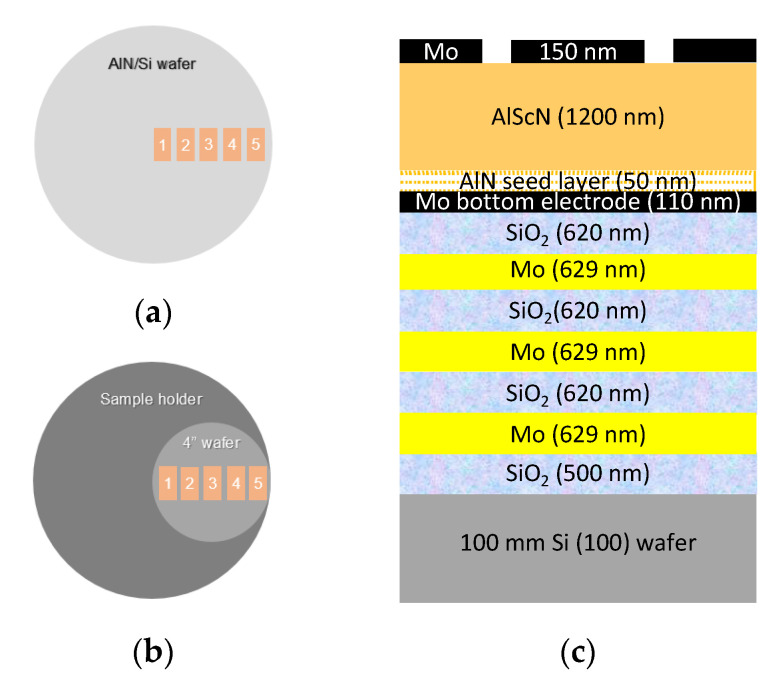
(**a**) Diagram of 8-inch AlN seed layer/Si wafer used as substrate. Numbers from 1 to 5 represent the spots where the samples were taken from. (**b**) Diagram of the disposition inside the sputtering chamber of the 4-inch wafer used to fabricate the SMRs. (**c**) Cross section of the manufactured SMRs.

The AlScN films were deposited on two types of substrates. To assess the structural properties and composition of AlScN sputtered films, 720 nm thick AlScN films were deposited on 8-inch Si wafers covered with a 50 nm thick AlN seed layer deposited without breaking vacuum (see Figure 2a). As one of the most important aspects of our study was to evaluate uniformity in properties throughout the films, 5 samples were taken from equidistant positions following the wafer radius. These samples were used to perform RBS+NRBS and XRD measurements, before and after applying a 600 °C thermal treatment in a vacuum for 1 h, as will be detailed below. Extra samples with the same characteristics were also annealed at 700 °C and used to obtain extra XRD measurements.

To assess the piezoelectric activity of the films, 4-inch Si wafers (Figure 2b) were used to manufacture SMRs excited with the AlScN piezoelectric films. A 7-layer acoustic reflector was first fabricated on top of the Si substrate by alternating 620 nm thick SiO_2_ films (low acoustic impedance layers) and 629 nm thick Mo films (high impedance layers) to confine the acoustic energy. The piezoelectric sandwich manufactured on top of the mirror consisted of a 110 nm thick Mo bottom electrode, the 50 nm AlN seed layer followed by the AlScN piezoelectric film (1.1 µm), and a 150 nm thick Mo top electrode, as shown in Figure 2c. The Mo top electrode was finally patterned to excite the piezoelectric film via capacitive coupling. Figure 2b shows the wafer disposition within the sputtering chamber and the spots where the 5 samples were taken from to evaluate film uniformity in terms of piezoelectric properties, before and after applying 600 °C annealing in a vacuum for 1 h. It is worth noting that these spots are practically the same as the ones chosen for the 8-inch wafers displayed in Figure 2, as this leaves us the possibility of establishing correlations between the results obtained in all the characterization techniques applied in this study.

## 3. Results and Discussion

Al_0.7_Sc_0.3_N films were characterized before and after applying a thermal treatment of 600 °C for 1 h in a vacuum to assess their thermal stability in terms of their piezoelectric and structural properties.

### 3.1. SMR Electrical Response

**Figure 3:**
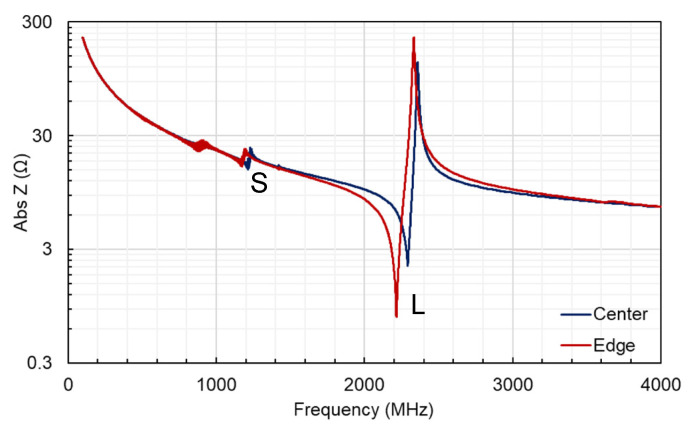
Frequency response of Al_0.7_Sc_0.3_N-based SMRs located at position corresponding to the sample holder center and located at sample holder edge. Shear and longitudinal modes are labeled with an S and L, respectively.

The frequency response of the SMRs fabricated on the 4-inch Si wafers was assessed by measuring the S_11_ reflection coefficient using a vector network analyzer and then obtaining the impedance and admittance spectra. Figure 3 shows an example of an impedance spectrum from two resonators: one located at the right edge of the 4″ wafer, corresponding to the edge of sample holder, and another one located at the left edge of the 4″ wafer, corresponding to the center of the sample holder. The c-axis favoring the growth of Al_0.7_Sc_0.3_N films is evident after observing the longitudinal mode located at around 2 GHz. However, a shear mode is also observed at around 1.3 GHz, suggesting some dispersion in the microcrystal orientation along the c-axis, although this is not very significant since 10·1, 10·2 or 10·3 peaks are not observed in the XRD patterns, as discussed in the next section. It is worth noting that the shear mode is excited in both samples, although generally, it appears to be of slightly higher intensity at the center of the wafer. This might be related to an increased deviation of the c-axis-oriented microcrystals, possibly related to the presence of some structural defects in this area.

**Figure 4:**
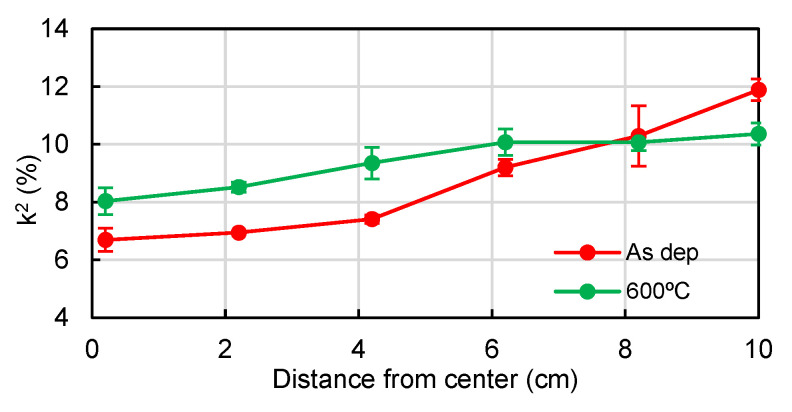
Radial distribution of mean k^2^ values for Al_0.7_Sc_0.3_N-based SMRs. The distance is measured from the sputtering chamber sample holder center. The error bars represent one standard deviation.

The electromechanical coupling factor (k^2^) of a series of SMRs of different shapes and sizes derived from the S_11_ measurements. This parameter is almost exclusively dependent of the piezoelectric material and can be used to estimate whether the Al_0.7_Sc_0.3_N films display high quality in terms of their piezoelectric properties. Figure 4 shows the k^2^ radial distribution for different wafer spots before and after the thermal treatment. The k^2^ values appearing on the chart represent the mean value of the distribution of the resonators located in each position. The error bars attached to each point represent one standard deviation from each k^2^ distribution. As one might observe, the k^2^ values increase as the distance from the center increases, revealing an improvement in the piezoelectric activity of the films. This could be related to a difference in film homogeneity derived from the sputtering process. Despite this difference in electromechanical coupling, most of the resonators show a better k^2^ value than the usual ones obtained from AlN-based resonators, which usually range between 6% and 7% [23,24,25]. In this case, k^2^ values all range from 6% to 12%, with 12.8% being the highest measured value. A comparison between state-of-the-art values is shown in Table 1. After the thermal treatment, resonators located near the central positions tend to improve their piezoelectric properties and their k^2^ coefficients increase to the 8–10% range. Regarding more external spots, the annealing seems to worsen the piezoelectric properties of this area, and thus their k^2^ became slightly worse, dropping to values above 10%.

In addition to these measurements, Table 2 shows the measured Q-factor distribution of the resonators located at the spots with lower (center) and higher (edges) electromechanical coupling factors. It is noticeable how they are quite similar to each other regardless of the wafer position for both resonant and antiresonant frequencies. These values remain similar to each other after the annealing treatment, which might indicate that no extra acoustic losses are induced.

### 3.2. SEM and AFM Characterization

**Figure 5:**
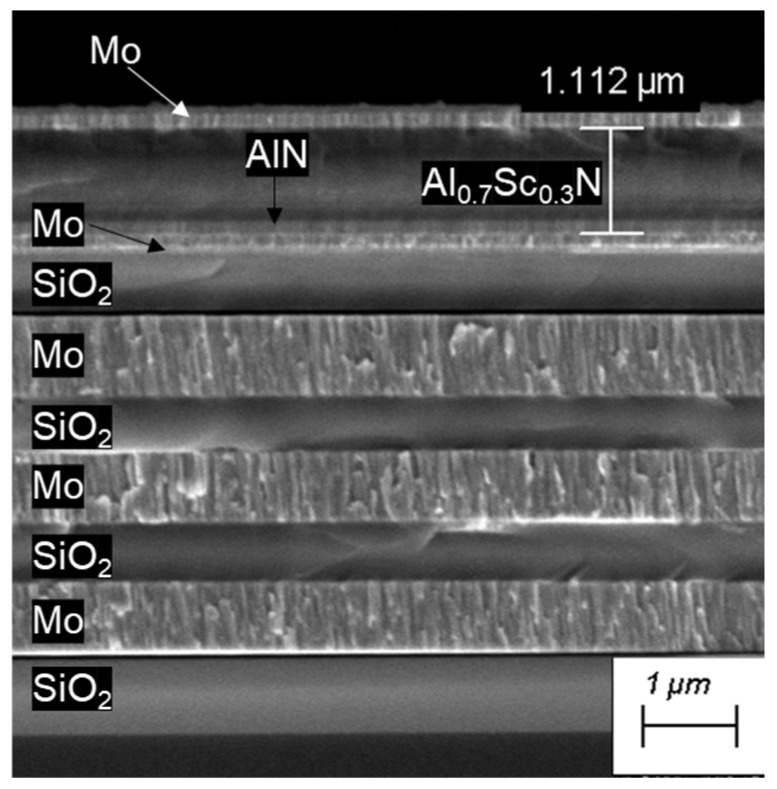
Cross-sectional SEM micrograph of the SMR stack.

Morphological characterization was carried out via the SEM technique. The alternating Mo and SiO_2_ showed distinct and clear interfaces. They also showed good thickness uniformity, the same as the two Mo electrodes that sandwiched the piezoelectric films. The Al_0.7_Sc_0.3_N piezoelectric films are distinguishable from the AlN seed layers and display quite good uniformity in terms of thickness throughout the entire wafers. A typical micrograph of the cross section from a cleaved SMR sample can be seen in Figure 5.

**Figure 6:**
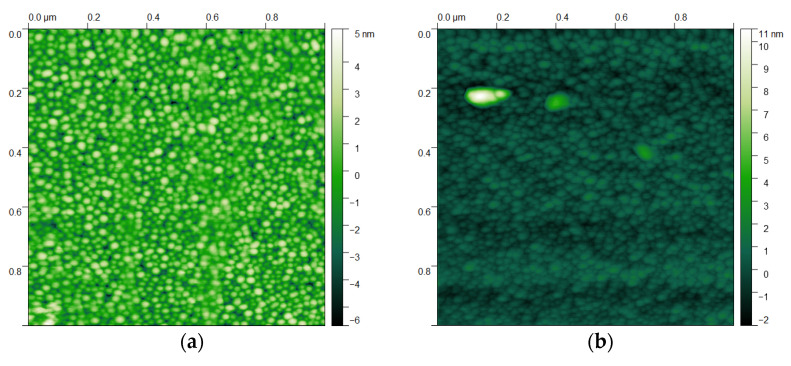
(**a**) AFM topographic image of the center and (**b**) the edge of the Al_0.7_Sc_0.3_N films deposited on 8-inch Si wafers.

The surface of the piezoelectric films was evaluated via AFM. Figure 6a shows a 1×1 μm topographic profile from the center of the wafer. A quite uniform and smooth surface can be observed, showing a root mean square roughness parameter of $R_{RMS}=1.34\;\mathrm{nm}$. Figure 6b shows the same measurement from a spot near the edge of the wafer. In this case, the presence of abnormal grains is spotted, with sizes up to ∼20 nm in height. However, the overall surface roughness improves, and calculating the $R_{RMS}$ parameter from the regions with no abnormal grains, the results are lower ($R_{RMS}=550\;\mathrm{pm})$.

### 3.3. Compositional Study Using RBS and NRA

The severe dispersion observed in the piezoelectric activity of the Al_0.7_Sc_0.3_N films across the wafer, as well the low thermal stability displayed by the films, prompted us to further examine the radial composition of the films across the 8-inch wafers through RBS and NRA measurements.

RBS measurements were performed in a 5 MeV tandem accelerator of the CMAM of the University Autónoma of Madrid. A first set of experiments which was already published in [22] was performed using 4He+ ions accelerated at 2.3 MeV, impinging normally on the samples at a dose of 5 μC. The ions backscattered at  170° were measured with a solid-state surface barrier detector with a solid angle of 3.9 msr and a resolution of 20 keV. The fitting of the spectra using the SIMRA code [29] revealed that the thickness of the Al_0.7_Sc_0.3_N film was uniform across the 8” wafer and that the Al-to-Sc ratio in the films was relatively constant along the wafer radius, going from 28.4% at the center to 30.6% at the edge of the wafer (Table 3). However, this slight dispersion could not account for the variation in the piezoelectric behavior we observed in different film regions.

**Figure 7:**
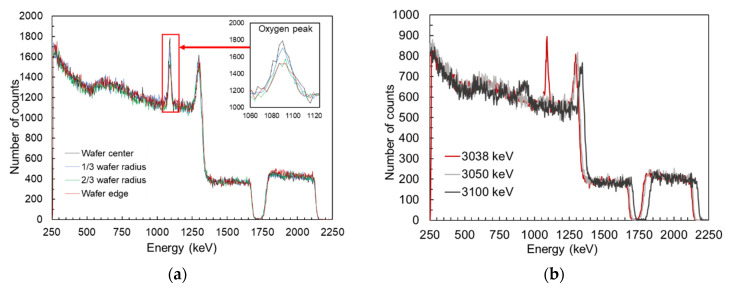
(**a**) NRBS spectra taken at different wafer positions, fixing the beam energy at 3.038 MeV. The inset highlights the oxygen resonance detected when measuring at this energy [34]. (**b**) RBS-NRA spectra from wafer center taken at three different beam energies.

Since RBS has better sensitivity to heavy elements than light elements, the presence of light impurities in the films (O, C) could have been easily disregarded in the first set of experiments, which is why a deeper look for impurities and/or other defects was needed. A second set of experiments was performed at higher energies at which the scattering cross section of light elements deviate from Rutherford cross sections (non-Rutherford backscattering) to take advantage of their significant increase at certain energies, called resonances [30]. First, we measured the NRBS spectra at the energy of 3.038 MeV, at which the scattering cross section of oxygen peaks is linked to the ^16^O(α, α)^16^O nuclear reaction. Figure 7a shows the NRBS spectra measured at 3.038 MeV on four different film spots, following a radial distribution. The almost identical patterns observed in the region between 1700 keV and 2250 keV (corresponding to the Sc signal) confirms the good homogeneity in terms of thickness of the Al_0.7_Sc_0.3_N film [31]. The inset of Figure 7a outlines the signal peaks corresponding to oxygen located at the surface of the films. The measurement shows that the oxygen content clearly increases when moving from the edges to the center of the films, which might suggest that the central region of the wafer is more susceptible to adsorb oxygen atoms than the external region. This effect might be caused by a larger ion bombardment of this area of the sample holder during deposition, thus producing a more deteriorated surface with a higher concentration of structural defects, such as dangling bonds, which could end up in an increase in surface reactivity and the capturing of more oxygen atoms than other regions [32,33].

To obtain more insight into the in-depth oxygen distribution along the Al_0.7_Sc_0.3_N film, two other measurements were performed at higher energies (3.05 MeV and 3.1 MeV) to verify whether projectiles backscattered at 3.038 MeV with oxygen atoms could come from greater depths in the sample. As can be seen in the resulting spectra shown in Figure 7b, no signal corresponding to backscattering with O atoms is observed for these energies, in contrast to what is observed at 3.038 MeV, suggesting that oxygen is only present at the surface of the film, as higher beam energies do not produce any peak related to the oxygen presence in the bulk of the material.

In addition to these measurements, other spectra were measured at 4.258 MeV, at which the scattering cross section of carbon (C) is maximized, owing to the ^12^C(α, α)^12^C nuclear reaction. These measurements did not reveal the presence of this impurity at the surface of the film.

In summary, the compositional characterization indicates that the bulk of the AlScN has a composition close to Al_0.7_Sc_0.3_N and is free of O and C. However, some adsorbed O can be found at the surface of the film, more likely in the central area of the wafer, which is attributed to an increased surface reactivity caused by excessive ion bombardment during deposition.

### 3.4. Structural Study: XRD Measurements

X-ray diffraction (XRD) measurements were performed on the Al_0.7_Sc_0.3_N films deposited on the 8-inch Si wafers to obtain a deeper understanding of their crystalline structure across the whole wafer. Theta-2theta measurements and rocking curve (RC) around the main peaks were taken from different equidistant spots following the wafer radius, using an X’PERT PRO-MRD high resolution diffractometer provided with a Cu anode. After thermal treatment, the process was repeated on the same spots to obtain new XRD patterns to compare them with the previous ones.

**Figure 8:**
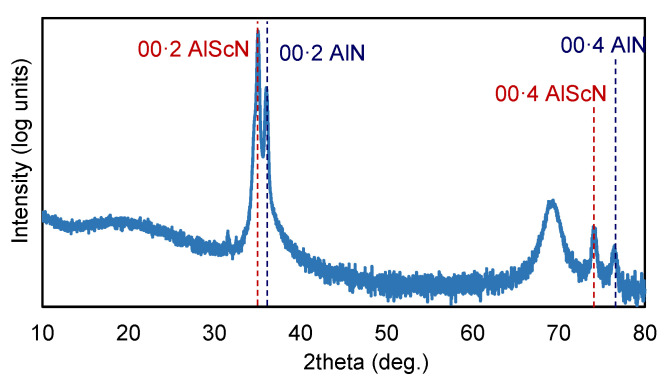
Typical XRD pattern of Al_0.7_Sc_0.3_N/AlN/Si structure in log units to highlight the absence of 10·1, 10·2 and 10·3 peaks.

**Figure 9:**
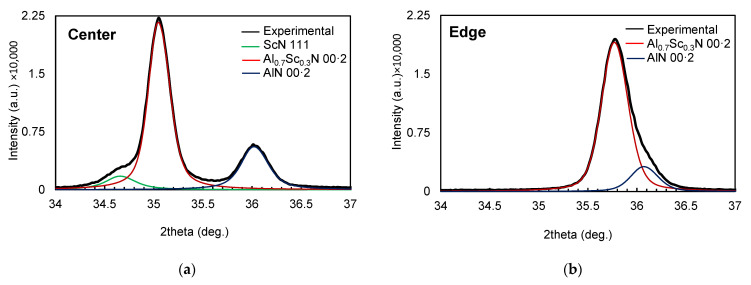
(**a**) Deconvoluted XRD pattern for a sample located at the center and (**b**) at the edge of the wafer.

As can be observed in Figure 8, typical theta-2theta patterns only displayed the 00·2 (and 00·4) reflections corresponding to both Al_0.7_Sc_0.3_N films and the AlN seed layer, with no evidence of misoriented grains, as 10·1, 10·2 and 10·3 peaks are not observed. Figure 7 also shows that the region gathering the most information about the structure of the films is located in the range between 34° and 37° in 2θ. Figure 8 shows this region in more detail, together with the resulting pseudo-Voigt functions used to perform the deconvolution of the main features for two samples located at the center (Figure 9a) and the edge (Figure 9b) of the wafer. 

The main features between 34° and 37° can be fitted by using three pseudo-Voight functions in the sample located at the center of the wafer, whereas only two functions are required to deconvolute the peaks at the edge of the wafer. The peak at around 36° present in the two samples is attributed to the 00·2 AlN peak of the seed layer. The main peak of the two spectra is associated with the reflection with the 00·2 planes of the Al_0.7_Sc_0.3_N film; the fact that the peak experiences a significant shift between samples could be related to differences in planar stress, since RBS measurements allows us to rule out peak shifts due to compositional changes. An extra peak at ~34.5° is required to fit the features at the central area of the wafer, which could be attributed to the non-piezoelectric 111 ScN rocksalt phase [35]. The presence of such a phase in the central area of the wafer is apparently related to the poorer piezoelectric response revealed by the SMR’s frequency response as well as to the increased surface reactivity revealed by the RBS-NRBS compositional characterization.

**Figure 10:**
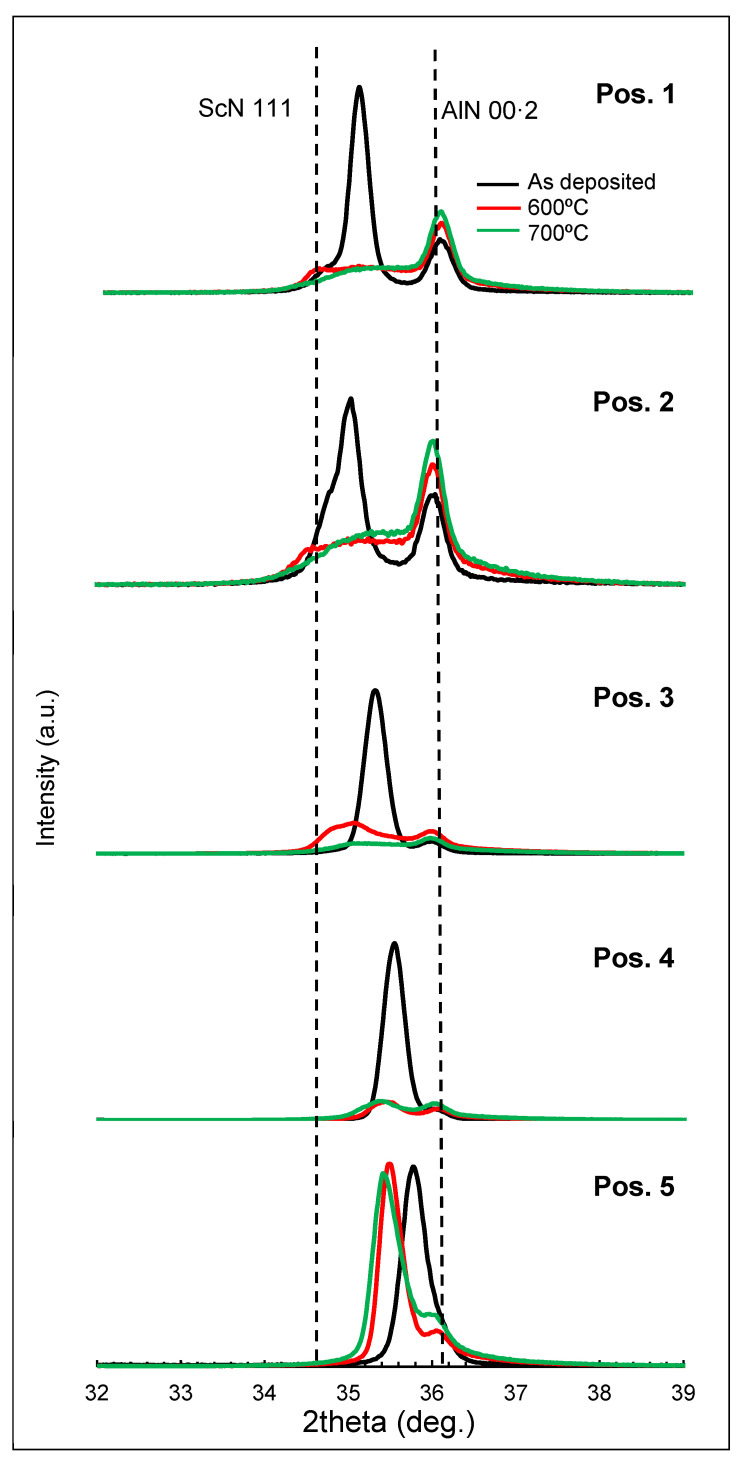
XRD patterns from the Al_0.7_Sc_0.3_N on Si films as deposited and after 600 °C and 700 °C thermal treatments, going from the center (Pos. 1) to the edge (Pos. 5) of the 8-inch wafer.

In order to gain more insight about the structural homogeneity and thermal stability of the Al_0.7_Sc_0.3_N films, XRD measurements were performed on five samples taken from equidistant positions along the wafer radius, before and after annealing at 600 °C and 700 °C. The theta-2 theta patterns of Figure 10 led to the following conclusions. (1) The AlN seed layer is indeed homogeneous along the wafer and exhibits high thermal stability. (2) The ScN rocksalt phase is apparently present in all the central areas (up to position 3), although the peak deconvolution does not allow us to draw significant conclusions about its thermal stability. (3) The shift of the 00·2 Al_0.7_Sc_0.3_N towards higher angles is confirmed, suggesting a stress gradient along the radius of the wafer in the as-deposited samples. When bringing the thermal treatments under discussion, the XRD patterns clearly reveal considerable changes affecting the 00·2 Al_0.7_Sc_0.3_N peak. A severe reduction in the peak intensities accompanied by a widening of the full width at half maximum (FWHM) is observed. Peak deconvolution after the heat treatments is not straightforward: the significant flattening of the main features after the heat treatments in all positions except in external areas might suggest an in-depth stress gradient in the Al_0.7_Sc_0.3_N film and/or a decrease in the crystallite size. The shift to lower 2-theta angles is also observed in similar recent investigations of 00·2 Al_0.7_Sc_0.3_N peaks after thermal treatments [36].

The RCs of the Al_0.7_Sc_0.3_N and AlN peaks were also measured and fitted using gaussian functions to obtain their FWHM for the as-deposited films and after 600 °C annealing. The results are gathered in Table 4, together with the calculated corresponding standard errors. The RC around the AlN 00·2 peak shows minor variations across the radius of the wafer, keeping an almost constant value after the annealing. Concerning the rest of the RCs at the wafer center, no clear pattern is observed. This could reinforce the idea that relevant structural changes occur once the thermal treatment is applied and that for this film region, the deposited material lacks stability and homogeneity. A similar behavior is observed for RCs in the mid-radius region. However, the fitted values for the external region reveal that the Al_0.7_Sc_0.3_N RCs remain similar, implying that the crystallites of this region could have a more stable structure.

In summary, the results of the composition and structural characterization might help explain, in part, the piezoelectric behavior observed in Figure 4. As RBS-NRA results suggested first, the excessive bombardment during the deposition phase might have produced Al_0.7_Sc_0.3_N films with a large number of structural defects in central regions. This, together with the presence of an extra XRD peak related to a ScN non-piezoelectric phase, may explain the poor k^2^ values obtained when characterizing the fabricated SMRs. This hypothesis might be well supported by the XRD patterns in Figure 10 in which one can observe the instability of these regions after applying thermal treatments. On the other hand, external regions seem to have fewer structural defects and a more stable crystalline structure, which could be the main reason for the good k^2^ values obtained. However, it is worth noting that structural changes are also observable in these regions as well, since these values slightly drop after thermal treatment.

## 4. Conclusions

Al_0.7_Sc_0.3_N sputtered thin films using Al-Sc targets and the consequences of applying post-deposition annealing were studied by measuring their composition, structural properties, and piezoelectric activity before and after the annealing.

We observe that, in terms of composition, the sputtering process achieves quite uniform thin films with consistent Sc content along the entire wafers. SEM micrographs and RBS spectra confirm good thickness uniformity for the deposited thin films. However, NRBS analysis suggests that central wafer positions might be subjected to greater ion bombardment during deposition, also supported by differences in surface roughness observed from AFM topography images. This is very likely correlated with the results observed in the XRD measurements, suggesting structural changes between regions, such as the coexistence of piezoelectric and non-piezoelectric phases in the central area of poor thermal stability, and a radial stress gradient in the Al_0.7_Sc_0.3_N film. All these findings might explain the highly variable piezoelectric activity distribution, with regions with poor k^2^ values measured from SMR devices. On the other hand, characterized devices from the external regions show very good piezoelectric behavior, with k^2^ values over 12%. After thermal treatments, no overall improvements are observed in either crystalline structure or piezoelectric activity. After these observations, we conclude that special attention should be paid to the structure of sputtered ternary AlScN and that a study of the thermal stability is essential for films intended to operate under harsh environments.

## Figures and Tables

**Table 1:** k^2^ values of best and worst resonator presented in this work in comparison with state-of-the-art FBAR devices.

Piezoelectric Material	$k^{2}$	Reference
AlN	6.5	[24]
Al_0.91_Sc_0.9_N	9.53	[12]
AlN/Al_0.87_Sc_0.13_N	10	[26]
Al_0.85_Sc_0.15_N	12	[12]
Al_0.8_Sc_0.2_N	14.5	[27]
Al_0.73_Sc_0.27_N	12.18	[28]
Al_0.7_Sc_0.3_N	6.3	This work (worst resonator)
Al_0.7_Sc_0.3_N	12.8	This work (best resonator)

**Table 2:** Measured Q_r_ and Q_a_ distributions.

Distance from Center (cm)	$Q_{r}\;\pm \;\sigma$	$Q_{a\;}\pm \;\sigma$
10 (As dep)	210±68	181±38
10 (after 600 °C)	176±78	200±31
0.2 (As dep)	205±107	194±18
0.2 (after 600 °C)	166±77	185±40

**Table 3:** RBS atomic composition at different wafer spots.

Distance from Center (cm)	Sc (%)	Al (%)	N (%)	$\frac{\mathrm{Sc}}{\mathrm{Al}+\mathrm{Sc}}\;(\%)$
0–2 cm	14.3	36	49.7	28.4
2–4 cm	14.5	35.8	49.7	28.8
4–6 cm	14.2	35.2	50.3	28.7
6–8 cm	14.8	33.5	51.6	30.6
8–10 cm	15.2	34.5	50.2	30.6

**Table 4:** FWHM values for the different RC measurements.

Al_0.7_Sc_0.3_N-00·2 Peak		
Distance from center (cm)	FWHM As dep.	FWHM @600 °C
0–2	2.09±0.01	3.04±0.02
4–6	1.78±0.01	2.68±0.01
8–10	1.32±0.01	1.35±0.01
AlN-00·2 peak		
Distance from center (cm)	FWHM As dep.	FWHM @600 °C
0–2	2.59±0.01	2.61±0.01
4–6	2.62±0.01	2.74±0.02
8–10	2.58±0.02	2.65±0.01

## Data Availability

Data are contained within the article.
